# HER2/neu and Ki-67 expression predict non-invasive recurrence following breast-conserving therapy for ductal carcinoma *in situ*

**DOI:** 10.1038/bjc.2012.41

**Published:** 2012-02-23

**Authors:** E Rakovitch, S Nofech-Mozes, W Hanna, S Narod, D Thiruchelvam, R Saskin, J Spayne, C Taylor, L Paszat

**Affiliations:** 1 Department of Radiation Oncology, University of Toronto, Toronto, Canada; 2 Department of Pathology, Sunnybrook Health Sciences Centre, University of Toronto, 2075 Bayview Avenue, Toronto M4N 3M5, Canada; 3 Institute of Clinical Evaluative Sciences, University of Toronto, Toronto, Canada; 4 Women's College Hospital Research Institute, University of Toronto, Toronto, Canada

**Keywords:** DCIS, biomarkers, recurrence, breast-conserving surgery

## Abstract

**Background:**

Ductal carcinoma *in situ* (DCIS) is a non-invasive form of breast cancer that may progress to invasive cancer. Identification of factors that predict recurrence and distinguish DCIS from invasive recurrence would facilitate treatment recommendations. We examined the prognostic value of nine molecular markers on the risks of local recurrence (DCIS and invasive) among women treated with breast-conserving therapy.

**Methods:**

A total of 213 women who were treated with breast-conserving therapy between 1982 and 2000 were included; 141 received breast-conserving surgery alone and 72 cases received radiotherapy. We performed immunohistochemical staining on the DCIS specimen for nine markers: oestrogen receptor, progesterone receptor, Ki-67, p53, p21, cyclinD1, HER2/neu, calgranulin and psoriasin. We performed univariable and multivariable survival analyses to identify markers associated with the recurrence.

**Results:**

The rate of recurrence at 10 years was 36% for patients treated with breast-conserving surgery alone and 18% for women who received breast-conserving surgery and radiotherapy. HER2/neu+/Ki-67+ expression was associated with an increased risk of DCIS recurrence, independent of grade and age (HR=3.22; 95% CI: 1.47–7.03; *P*=0.003). None of the nine markers were predictive of invasive recurrence.

**Conclusion:**

Women with a HER2/neu/neu+/Ki67+ DCIS have a higher risk of developing DCIS local recurrence after breast-conserving surgery.

Ductal carcinoma *in situ* (DCIS) is a non-invasive, highly treatable form of breast cancer. Ductal carcinoma *in situ* constitutes approximately 20% of breast cancers diagnosed through mammographic screening (Ernster *et al*, 2002). Following a diagnosis of DCIS, women are at elevated risk both for non-invasive (DCIS) recurrence and invasive local recurrence. Women who develop a local recurrence require additional treatment, such as further local excision or mastectomy, to reduce the risk of subsequent invasive breast cancer and potential breast cancer mortality (Solin *et al*, 1994; Silverstein *et al*, 1998).

Radiotherapy is highly effective in reducing the risk of local recurrence, but it requires a substantial time commitment from patients and carries potential side effects, including skin changes, cardiac morbidity and secondary malignancies (Fisher *et al*, 1998; Julien *et al*, 2000; Houghton *et al*, 2003; Paszat *et al*, 1998, 2007; Lauzier *et al*, 2011). At present, it is difficult to predict which women with DCIS will go on to develop recurrence following breast-conserving surgery for DCIS. Younger age at diagnosis and the presence of high-grade DCIS are associated with the development of local recurrence but these factors do not distinguish an individual's risk of developing DCIS from those at risk of invasive recurrence. As a result, most women diagnosed with DCIS receive radiotherapy following surgery.

In order to improve risk stratification and treatment recommendation for women with DCIS, there is a need to identify predictors of non-invasive local recurrence and invasive local recurrence following breast-conserving therapy. Several studies have evaluated the prognostic significance of biomarkers including oestrogen receptor (ER) expression, progesterone receptor (PR) expression and HER2/neu expression as predictors in DCIS but most of these studies were limited due to the number of patients included, variations in treatment and short follow-up (Ringberg *et al*, 2001; Provenzano *et al*, 2003; Cornfield *et al*, 2004). The largest study included a population-based cohort of women treated by breast-conserving surgery without radiotherapy. In this study, women with ER-/HER2/neu+/Ki67+ DCIS had a greater risk of developing DCIS recurrence than women with other expression profiles (Kerlikowske *et al*, 2010). However, only 33 cases in this study had this particular profile; therefore, we sought to validate their findings in our patient cohort. We evaluated the prognostic value of these markers and six additional biomarkers for the development of non-invasive (DCIS) and invasive breast cancer in women with DCIS treated by breast-conserving therapy.

## Patients and methods

### Patients

We reviewed the medical and pathology records of all 296 patients with a diagnosis of DCIS (alone or with microinvasion) who were treated at our institution between 1982 and 2000. Cases who were treated by mastectomy (*n*=42) were excluded from the analysis because there were no local recurrences and our objective was to evaluate biomarkers of recurrence following breast-conserving therapy. There were 254 cases treated by breast-conserving therapy, including 172 cases treated by breast-conserving surgery alone (*n*=172) and 82 cases treated by breast-conserving surgery followed by radiation. Tissue blocks were unavailable for 41 cases and these cases were excluded. Therefore, the study cohort includes 213 individuals, 141 cases treated by breast-conserving surgery alone and 72 cases treated by breast-conserving surgery and radiation. Data was obtained on age at diagnosis, clinical and pathological features and clinical outcomes, including the development of any local recurrence (DCIS or invasive), regional recurrence or distant recurrence. The presence of recurrent disease was validated by review of the pathology report. If the patient had not attended the cancer centre in the past 12 months, then follow-up information was obtained through correspondence with the primary physician and/or referring surgeon.

### Pathology

For each case of DCIS, all diagnostic slides and blocks were reviewed by an expert breast pathologist, using standardised criteria. For non-palpable lesions, the entire specimen was submitted and serially sectioned at 0.5 cm intervals (average 30 blocks per case). Specimen radiographs were taken and foci of calcifications, areas of architectural distortion and resection margins were identified, sectioned and submitted for evaluation. For specimens with a grossly identifiable mass, sections were submitted to evaluate the distance between the tumour and the resection margins. Overall, we examined an average of 12 blocks per case. Tumour size was evaluated as a continuous variable (cm). Nuclear grade was determined using the Holland classification (Holland *et al*, 1994) and categorised as low, intermediate and high. Lesions with mixed grade were coded as the highest grade observed. Comedo-necrosis was considered to be the presence of any architectural pattern of DCIS in which a central zone of necrotic debris with karyorrhexis was identified. Microinvasion was defined as the presence of invasive cancer measuring ⩽1 mm (patients with an invasive component of greater size were considered to be invasive cancers and were excluded)(Edge *et al*, 2010). The resection margin status was reported as positive when DCIS was present at the inked or cauterised edge of the specimen and negative if there was no DCIS at the inked margin. For some patients, the surgical cavity was re-excised in order to obtain a negative margin. The final margin status refers to the resection margin of the final surgical specimen. The width of the negative resection margin represents the closest distance of DCIS to the edge of the specimen and was categorised as ⩽3 mm, 4–9 mm, ⩾10 mm or unreported. Multifocality was defined as more than one distinct focus of DCIS, with at least 5 mm of intervening normal tissue (Sikand *et al*, 2005). For cases with multifocal DCIS, the size of the largest focus was recorded.

### Immunohistochemistry of molecular markers

We evaluated the expression of ER, PR, HER2/neu, Ki67, p53, p21, cyclin D1, calgranulin and psoriasin on formalin-fixed paraffin-embedded tissue sections, using immunohistochemistry (Table 1). A positive and negative control were included in every assay. The positive control for psoriasin and calgranulin consisted of two cases of DCIS with an invasive component. In these cases positive psoriasin and calgranulin immunostains correlated with their gene upregulation as shown using DNA microarray technique (Seth *et al*, 2003). We used the MIB1 antibody for evaluating Ki67 expression. Nuclear staining was scored for ER, PR, Ki67, p53, p21, cyclin D1, and membranous staining for HER2/neu oncoprotein, and both nuclear and cytoplasmic staining were scored for calgranulin and psoriasin. HER2/neu immunostain was scored from 0 to 3+ as per the HerceptTest scoring method. In equivocal cases, *HER2/neu* gene amplification was determined by chromogenic *in situ* hybridisation (CISH). This was performed using the Zymed SPoT-Light HER2 CISH Polymer Detection kit (84–0146; Zymed, San Francisco, CA, USA). *HER2/neu* gene amplification was determined when there were six or more signals per nucleus or when clusters were identified in the cell nuclei. For ER and PR, a 10% cutoff value was used to categorise cases into positive or negative. The results of the other immunohistochemical markers were recorded as continuous variables, based on the proportion of positive tumour cells (0–100%) regardless of staining intensity. The molecular markers psoriasin, calgranulin, p21, p53 and Ki67 do not have known cut points. Therefore, in addition to evaluating these markers as continuous variables, we compared the outcomes of cases with low immunostaining (<10%) to those with higher staining (⩾10%). One pathologist (SNM) evaluated all the immunostains. A second breast pathologist (WH) reviewed a sample of 20% of all the slides, as well as all slides where HER2/neu status was considered equivocal. The agreement between pathologists was greater than 90%. The pathologists were blinded to the patient outcomes. All treatments and outcomes were confirmed by review of pathology and/or operative reports.

### Statistical analysis

We performed a descriptive analysis to compare, for each of the nine markers, the proportion of patients who did and who did not recur. The differences in proportions for categorical variables were evaluated for significance using the *χ*^2^-test. Differences in means for continuous variables were evaluated for significance using Student's *t*-tests. We performed univariate and multivariate survival analyses using the Cox proportional hazards model to evaluate the associations between independent variables and the following outcomes: (1) any recurrence (local or invasive), (2) DCIS recurrence and (3) invasive recurrence. Local recurrence was defined as invasive cancer or DCIS that occurred in the ipsilateral breast, at least 6 months following the diagnosis of DCIS. We evaluated the association between the histological features of DCIS (tumour size, nuclear grade, the presence of multifocality, margin size and architectural subtype) and the outcomes. Tumour size was coded as the largest focus of DCIS (cm). Nuclear grade and the presence of comedo necrosis were not entered into the model simultaneously because they were highly correlated. Analyses were adjusted for the effect of age at diagnosis and radiation. The administration of radiation was the strongest predictor of recurrence and there were no significant interactions observed between radiation and histopathological features of DCIS for any of the outcomes. Therefore, we repeated the analyses and present hazard ratios in women treated by breast-conserving surgery alone.

We then conducted the analyses to evaluate the relationship between molecular biomarker expression and the outcomes. We calculated multivariable hazards ratios and their corresponding 95% confidence intervals (CI) initially for each marker alone and then for combinations of biomarkers. All multivariable analyses were adjusted for the effects of age at diagnosis and radiation. We also adjusted for the effect of nuclear grade because of reports that high grade DCIS may be more likely to over express HER2/neu (Latta *et al*, 2002). We calculated hazard ratios for psoriasin, calgranulin, Ki67, p53, cyclin D1 and p21 as continuous variables (to represent a change in the risk of recurrence per 10% change in cellular staining) and categorical variables, where positive represents ⩾10% cells stain positive. There was no difference in the results; therefore, categorical results are presented. Hazard ratios for HER2/neu, ER status and PR status represent a change in the risk for positive compared with the negative status. Patients were followed from the date of diagnosis until either the date of recurrence, death from another cause, loss to follow-up or the date of the last clinic visit (or physician report). No patient withdrew from the study. Research ethics board approval was obtained.

## Results

### Cohort characteristics and outcomes

We reviewed the medical records of 213 patients with DCIS. Among the 72 women treated with breast-conserving surgery and radiotherapy, there were eight recurrences (11%) (five DCIS and three invasive). Median time of follow-up was 7.7 years (range 0.32, 14.1 years). The actuarial rate of local recurrence was 5.9% at 5 years and was 17.6% at 10 years. The rate of DCIS local recurrence was 4.5% at 5 years and was 10.8% at 10 years. The rate of invasive local recurrence was 1.4% at 5 years and was 7.7% at 10 years. Among women who experienced a recurrence, the median time to recurrence was 4.1 years for any recurrence, 2.5 years for DCIS recurrence and was 5.7 years for invasive local recurrence.

Of the 141 patients treated with breast-conserving surgery alone, there were 42 recurrences (26%) including 3 distant recurrences. In all, 21 of the 42 local recurrences were invasive (50%). The characteristics of these patients are presented in Tables 1 and 2. The median time of follow-up for individuals treated by breast-conserving surgery alone was 8.7 years (range 0–16.2 years). The actuarial rate of local recurrence was 20% at 5 years and was 36% at 10 years. The rate of DCIS local recurrence was 14% at 5 years and was 18% at 10 years. The rate of invasive local recurrence was 7% at 5 years and was 22% at 10 years. Among women who experienced a recurrence, the median time to recurrence was 4.2 years for any recurrence, 2.6 years for DCIS recurrence and was 5.6 years for invasive local recurrence. Women treated by breast-conserving surgery alone were slightly older at diagnosis, had smaller lesions and less likely to have high grade DCIS than women treated by breast-conserving surgery and radiation (Table 1).

### Predictors of local recurrence

We performed univariable and multivariable analyses to evaluate the association between the histological features of DCIS and the development of any local recurrence, DCIS recurrence and invasive recurrence in women treated by BCS alone. The presence of high nuclear grade and multifocality were significantly associated with the development of any local recurrence. For high nuclear grade, the hazard ratio was 2.21 (95% CI: 1.14, 4.29, *P*=0.02) for any local recurrence and was 4.09 (95% CI: 1.49, 11.23, *P*=0.01) for DCIS recurrence. For multifocality, the hazard ratio was 2.09 (95% CI: 1.09, 4.01, *P*=0.03) for any local recurrence and was 2.66, (95% CI: 1.03, 6.88, *P*=0.04) for DCIS local recurrence. None of the histological features of DCIS predicted for the development of invasive recurrence (Table 2).

We calculated hazard ratios associated with each of the molecular markers (adjusting for the effect of radiotherapy and age at diagnosis); first for any local recurrence (non-invasive or invasive) and then for DCIS recurrence and invasive recurrence. (Table 3). HER2/neu overexpression was the only molecular marker associated with an increased risk of any local recurrence on univariable analysis (HR: 2.11, 95% CI: 1.21, 3.68, *P*=0.01).

**Figure 1:**
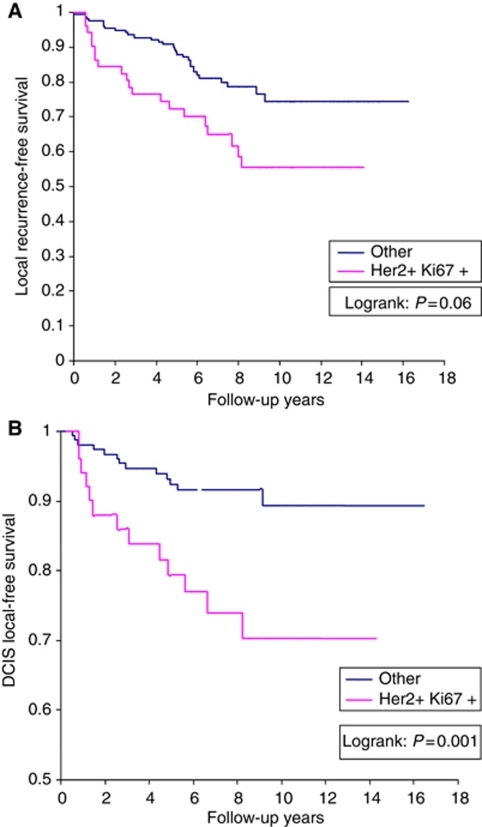
Her2/neu and Ki67 expression in DCIS is associated with the development of local recurrence (**A**) and DCIS recurrence (**B**).

In a univariable analysis, Ki-67 did not predict for local recurrence. However, on multivariable analysis, individuals with HER2neu+/Ki-67+ DCIS had a high likelihood of developing local recurrence. The 10-year rate of local recurrence was 39% (20/51) among HER2/neu+/Ki-67+ cases of DCIS compared with 18.5% (30/162) for cases without this profile (*P*=0.0024). The hazard ratio for any local recurrence was 2.15 (95% CI: 1.20–3.83, *P*=0.01), compared to women with other profiles. On multivariable analysis, individuals with HER2/neu+/Ki-67+ profile were three times more likely to develop a non-invasive recurrence (HR=3.22, 95% CI: 1.47, 7.03, *P*=0.003) compared with cases with other molecular phenotypes. The effect of the expression profile was independent of nuclear grade (Figure 1).

Although the rate of DCIS recurrence was higher for cases with HER2/neu+/Ki67+ DCIS (13/51 (26%) compared with 13/162 (8%) for cases without this profile, *P*=0.0009), we could not determine the effect of Ki67 positivity in the presence of HER2/neu positivity, because the majority (51/58) of HER2/neu+ cases were Ki67+. Among the seven cases with HER2/neu+/Ki-67− DCIS, none developed a DCIS recurrence and two developed an invasive recurrence. None of the molecular markers, either alone or in combination, were associated with an increased risk of invasive recurrence (Tables 4 and 5).

## Discussion

Our findings corroborate the results of a study by Kerlikowske *et al* (2010) of a population-based cohort of individuals with DCIS treated by breast-conserving surgery alone. In this earlier study, DCIS with HER2/neu+/Ki67+ expression was associated with a higher risk of DCIS recurrence (univariate HR=1.9, 95% CI: 1.0–3.5) but was not associated with the development of invasive recurrence. In the present study, the two-marker profile was also associated with a high risk of DCIS recurrence (multivariate HR=3.22, 95% CI: 1.47, 7.03, *P*=0.003) and not with invasive recurrence.

The effect of HER2/neu/Ki67 positivity was independent of nuclear grade. In contrast to the Kerlikowske study, we did not find that the addition of ER status to the two-marker profile added additional predictive information. The differences between the two studies may be due to the differences in patient populations, the precision of the hazards ratio estimate or to differences in the coding of HER2/neu positivity. Both studies coded DCIS lesions with a score of 3+ on immunostaining for HER2/neu as HER2/neu positive but DCIS lesions with equivocal immunostaining for HER2/neu (score of 2+) were scored as positive in the Kerlikowske study. We performed *in situ* hybridisation on all equivocal cases and only lesions with amplification were coded as HER2/neu+.

We studied a total of nine molecular markers for their ability to predict the development of recurrence following treatment of DCIS with breast-conserving therapy in a large cohort with long-term follow-up interval. We systematically evaluated histopathological features of DCIS and molecular biomarkers associated with the development of any local recurrence, DCIS local recurrence or invasive local recurrence. None of the markers was predictive of invasive recurrence.

Overall, a third (31%) of women with HER2/neu+/Ki67+ DCIS developed local recurrence following breast-conserving therapy compared to 16% (26/162) with different biomarker profiles. Among women who were not treated with radiotherapy, the risk associated with this profile was 47%. Among 17 women with this profile, who were treated with breast-conserving surgery and radiation, (24%) developed a local recurrence. This data suggests that women with HER2/neu+/Ki67+ DCIS are not optimal candidates for the treatment by breast-conserving surgery alone. Further research in other large cohorts is needed to evaluate the long-term outcomes of women with HER2/neu+/Ki67+ DCIS treated with BCS and radiation.

The identification of molecular predictors in DCIS is challenging because the number of recurrences is low and the expression levels of molecular biomarkers are often correlated (Latta *et al*, 2002). This limits our ability to evaluate multiple markers simultaneously in a multivariable model. Cut point values for many molecular markers have not been established. Furthermore, systematic pathological and molecular evaluation and long-term follow-up is required. As a result, most studies have been limited in statistical power and have not differentiated biomarker profiles associated with invasive recurrences and non-invasive recurrences (Ringberg *et al*, 2001; Provenzano *et al*, 2003; Cornfield *et al*, 2004). However, as we show here, the molecular alterations associated with DCIS local recurrence differ from those associated with invasive recurrence. It is important to distinguish these two endpoints. Further research is needed to identify biomarkers that distinguish individuals at risk of DCIS recurrence from those at risk of developing invasive recurrence, to further evaluate the effect of joint expression of HER2/neu and Ki67 and of other combinations of biomarkers on the risks of local and invasive recurrence and to evaluate the effect of radiation on these outcomes.

## Figures and Tables

**Table 1:** Characteristics of patients with DCIS in the study

	**Breast-conserving surgery+radiotherapy (*N*=72)**	**Breast-conserving surgery (*N*=141)**	***P*-value**
*Age mean* (range) (years)	54.4 (33.4, 81.6)	58.1 (27, 86)	0.04
			
*Tumour size (cm)*			
Mean (range)	1.2 (0.09, 5.0)	0.8 (0.02, 2.5)	<0.001
			
*Comedo necrosis*			
Yes	48 (67%)	87 (62%)	0.48
No	20 (28%)	53 (37%)	
Missing	4 (5%)	1 (1%)	
			
*Nuclear grade*			
Low	5 (7%)	20 (14%)	0.034
Moderate	27 (38%)	65 (46%)	
High	38 (53%)	56 (40%)	
Missing	2 (2%)		
			
*Multifocality*			
Yes	33 (46%)	50 (35%)	0.14
No	39 (54%)	91 (65%	
			
*Microinvasion*			
Yes	4 (6%)	4 (3%)	0.32
No	68 (94%)	137 (97%)	
			
*Size of negative margin (mm)*			
⩽3	40 (56%)	78 (61%)	0.61
4–9	10 (14%)	22 (17%)	
⩾10	19 (27%)	24 (19%)	
Missing	2 (3%)	4 (3%)	
			
*Molecular markers*			
HER2/neu+	19 (26.4%)	39 (27.8%)	0.84
Psoriasin *continuous*	7.9 (0 ,100)	5.2 (0, 90)	0.27
Psoriasin (⩾10%)	18 (25.0%)	20 (14.2%)	0.051
Calgranulin *continuous*	5.2 (0, 75)	11.1 (0, 100)	0.06
Calgranulin (⩾10%)	11 (15.3%)	31 (22.0%)	0.24
Ki67 *continuos*	12.9 (0, 80)	13.4 (0, 80)	0.82
Ki67 (⩾10%)	49 (68.1%)	91 (64.5%)	0.61
p53–*continuous*	41.9 (0, 100)	15.1 (0, 100)	<0.001
p53+(⩾10%)	44 (61.1%)	39 (27.7%)	<0.001
ER positive	59 (81.9%)	94 (66.7%)	0.02
PR positive	52 (72.2%)	83 (58.9%)	0.06
Cyclin D1	71.5 (0, 100)	78.8 (0, 100)	0.047
p21–*continuous*	20.1 (0, 100)	20.1 (0, 100)	0.99
p21+(⩾10%)	41 (57.0%)	74 (52.4%)	0.54

**Table 2:** Histological predictors of local recurrence following breast-conserving surgery alone for DCIS: multivariable analysis (adjusted for age at diagnosis)

**Outcome**	**HR (95% CI)**	***P*-value**
*Any local recurrence*		
High nuclear grade	2.21 (1.14, 4.29)	0.02
Multifocality	2.09 (1.09, 4.01)	0.03
Tumour size	1.01 (0.94, 1.07)	0.89
Margin size	0.96 (0.86, 1.06)	0.40
Architectural subtype (solid *vs* other)	0.92 (0.45, 1.86)	0.82
		
*DCIS recurrence*		
High nuclear grade	4.09 (1.49, 11.23)	0.01
Multifocality	2.66 (1.03, 6.88)	0.04
Tumour size	1.04 (0.95, 1.13)	0.42
Margin size	1.01 (0.87, 1.16)	0.95
Architectural subtype (solid *vs* other)	1.35 (0.45, 4.03)	0.59
		
*Invasive recurrence*		
High nuclear grade	1.27 (0.49, 3.32)	0.62
Multifocality	1.66 (0.66, 4.17)	0.28
Tumour size	0.97 (0.88, 1.07)	0.54
Margin size	0.92 (0.79, 1.07)	0.28
Architectural subtype (solid *vs* other)	0.66 (0.26, 1.72)	0.40

**Table 3:** Molecular predictors of any local recurrence

**Variables**	***N***	**No of LR**	**Hazard ratio (95% CI)**	***P*-value**
*Univariable analysis*				
HER2/neu+			2.11 (1.21, 3.68)	0.01
Psoriasin (⩾10%)			0.81 (0.38, 1.72)	0.58
Calgranulin			1.35 (0.72, 2.54)	0.35
Ki67 (⩾10%)			0.91 (0.51, 1.61)	0.75
p53+(⩾10%)			0.89 (0.49, 1.59)	0.68
ER positive			0.85 (0.47, 1.53)	0.59
PR positive			0.94 (0.53, 1.65)	0.82
Cyclin D1			1.00 (0.99, 1.01)	0.74
p21+(⩾10%)			1.04 (0.59, 1.81)	0.90
				
*Multivariable analysis (adjusted for age and XRT)*				
Her2/neu positive (*vs* other)	58	22	2.10 (1.19, 3.69)	0.01
HER2/neu+/Ki67+ (*vs* other)	51	16	2.15 (1.20, 3.83)	0.01
HER2/neu+/Ki67− (*vs* other)	7	2	1.22 (0.29, 5.06)	0.79
HER2/neu+/p53+(*vs* other)	35	8	1.29 (0.64, 2.62)	0.48
Ki67+/p53+ (*vs* other)	63	12	1.23 (0.65, 2.33)	0.53
HER2/neu+/Ki67+/p53+ (*vs* other)	31	8	1.50 (0.73, 3.07)	0.27
ER−/HER2/neu+/Ki67+ (*vs* other)	31	11	1.52 (0.77, 2.99)	0.23

**Table 4:** Molecular predictors of non-invasive (DCIS) local recurrence

	***N***	**No of DCIS LR**	**Hazard ratio (95% CI)**	***P*-value**
*Univariable analysis*				
HER2/neu+			2.72 (1.26, 5.88)	0.01
Psoriasin (⩾10%)			1.30 (0.52, 3.24)	0.57
Calgranulin (⩾10%)			1.47 (0.62, 3.49)	0.39
Ki67 (⩾10%)			1.05 (0.47, 2.35)	0.91
p53 (⩾10%)			0.89 (0.40, 1.99)	0.77
ER positive			1.14 (0.48, 2.71)	0.77
PR positive			0.71 (0.33, 1.53)	0.37
Cyclin D1 (⩾10%)			1.01 (0.99, 1.02)	0.52
p21 (⩾10%)			1.24 (0.57, 2.71)	0.58
				
*Multivariable analysis (adjusted for age and XRT)*				
Her2/neu+	58	13	2.67 (1.23, 5.79)	0.01
HER2/neu+/Ki67+ (*vs* other)	51	10	3.22 (1.47, 7.03)	0.003
HER2/neu+/Ki67− (*vs* other)	7	0	Not calculable	
HER2/neu+/p53+(*vs* other)	35	5	1.54 (0.61, 3.91)	0.36
Ki67+/p53+ (*vs* other)	63	6	1.09 (0.44, 2.67)	0.86
HER2/neu+/Ki67+/p53+ (*vs* other)	31	5	1.79 (0.70, 4.57)	0.22
ER−/HER2/neu+/Ki67+ (*vs* other)	31	6	1.65 (0.66, 4.15)	0.28

**Table 5:** Molecular predictors of invasive recurrence

**Univariable analysis**	***N***	**No of Inv LR**	**Hazard ratio (95% CI)**	***P*-value**
HER2/neu+			1.58 (0.69, 3.62)	0.28
Psoriasin (⩾10%)			0.38 (0.09, 1.60)	0.19
Calgranulin (⩾10%)			1.24 (0.49, 3.12)	0.65
Ki67 (⩾10%)			0.79 (0.35, 1.77)	0.56
p53 (⩾10%)			0.88 (0.38, 2.06)	0.77
ER positive			0.64 (0.29, 1.45)	0.29
PR positive			1.30 (0.55, 3.03)	0.55
Cyclin D1 (⩾10%)			0.99 (0.98, 1.01)	0.85
p21 (⩾10%)			0.85 (0.38, 1.90)	0.69
				
*Multivariable analysis (adjusted for age and XRT*)				
HER2/neu/neu+	58	9	1.61 (0.70, 3.73)	0.26
HER2/neu+/Ki67+ (*vs* other)	51	6	1.33 (0.54, 3.28)	0.54
HER2/neu+/Ki67− (*vs* other)	7	2	1.22 (0.29, 5.06)	0.79
HER2/neu+/p53+(*vs* other)	35	3	1.04 (0.35, 3.11)	0.94
Ki67+/p53+ (*vs* other)	63	6	1.41 (0.57, 3.52)	0.46
HER2/neu+/Ki67+/p53+ (*vs* other)	31	3	1.22 (0.40, 3.69)	0.73
ER−/HER2/neu+/Ki67+ (*vs* other)	31	5	1.39 (0.51, 3.78)	0.52
